# FSTL1 aggravates cigarette smoke-induced airway inflammation and airway remodeling by regulating autophagy

**DOI:** 10.1186/s12890-021-01409-6

**Published:** 2021-01-28

**Authors:** Ying Liu, Jiawei Xu, Tian Liu, Jinxiang Wu, Jiping Zhao, Junfei Wang, Minfang Zou, Lili Cao, Xiaofei Liu, Yun Pan, Siyuan Huang, Liang Dong

**Affiliations:** 1 Department of Respiratory, Shandong Qianfoshan Hospital, Cheeloo College of Medicine, Shandong University, Jinan, China; 2 Department of Pulmonary Diseases, North Hospital, Baotou, Inner Mongolia China; 3 Department of Respiratory and Critical Care Medicine, Qilu Hospital, Jinan, China; 4 Department of Respiratory, Shandong Provincial Qianfoshan Hospital, Shandong University, The First Affiliated Hospital of Shandong First Medical University, Shandong Institute of Respiratory Diseases, #16766, Jingshi Road, Jinan CityShandong Province, 250014 China

**Keywords:** Follistatin-like protein-1, 3-methyladenine, Autophagy, Chronic obstructive pulmonary disease

## Abstract

**Background:**

Cigarette smoke (CS) is a major risk factor for Chronic Obstructive Pulmonary Disease (COPD). Follistatin-like protein 1 (FSTL1), a critical factor during embryogenesis particularly in respiratory lung development, is a novel mediator related to inflammation and tissue remodeling. We tried to investigate the role of FSTL1 in CS-induced autophagy dysregulation, airway inflammation and remodeling.

**Methods:**

Serum and lung specimens were obtained from COPD patients and controls. Adult female wild-type (WT) mice, FSTL1^±^ mice and FSTL1^flox/+^ mice were exposed to room air or chronic CS. Additionally, 3-methyladenine (3-MA), an inhibitor of autophagy, was applied in CS-exposed WT mice. The lung tissues and serum from patients and murine models were tested for FSTL1 and autophagy-associated protein expression by ELISA, western blotting and immunohistochemical. Autophagosome were observed using electron microscope technology. LTB4, IL-8 and TNF-α in bronchoalveolar lavage fluid of mice were examined using ELISA. Airway remodeling and lung function were also assessed.

**Results:**

Both FSTL1 and autophagy biomarkers increased in COPD patients and CS-exposed WT mice. Autophagy activation was upregulated in CS-exposed mice accompanied by airway remodeling and airway inflammation. FSTL1^±^ mice showed a lower level of CS-induced autophagy compared with the control mice. FSTL1^±^ mice can also resist CS-induced inflammatory response, airway remodeling and impaired lung function. CS-exposed WT mice with 3-MA pretreatment have a similar manifestation with CS-exposed FSTL1^±^ mice.

**Conclusions:**

FSTL1 promotes CS-induced COPD by modulating autophagy, therefore targeting FSTL1 and autophagy may shed light on treating cigarette smoke-induced COPD.

## Background

Chronic obstructive pulmonary disease(COPD), one of the life-threatening respiratory system disorders, is projected to become the third most common cause of death worldwide by 2030 [1–4]. The most recent prevalence of COPD in China is 13.7% in people aged 40 or older [5]. COPD is characterized by a sustained airflow limitation and emphysema associated with airway remodeling and inflammation [6]. Complicated innate factors (e.g. host factors) and extrinsic factors (e.g. cigarette smoking) contribute to COPD, and cigarette smoke exposure still represents the most important risk factor [7]. Most studies showed that cigarette smoke was the major cause of pathogenesis and progression of COPD, which resulted in tremendous intractable inflammation and oxidative burden [8]. However, the underlying mechanisms of COPD induced by cigarette smoke are complex and not fully understood [9]. So far, there are no accurate therapies that can effectively retard or reverse disease progression.

Autophagy is a process of self-cannibalization of cellular components and a powerful promoter of metabolic homeostasis, which was activated by oxidative stress or the accumulation of damaged proteins or organelles [10, 11]. The elongation and maturation of autophagosomes require two ubiquitin-like conjugation systems: the microtubule-associated protein 3 light chain (lc3-atg8) and Atg5-Atg12-Atg16L conjugation systems [12]. Numerous studies report that autophagy plays an essential role either pathogenic or therapeutic in disease progression, especially in the inflammatory response in chronic respiratory disease [9, 13, 14]. Previous studies indicated that CS induced autophagy activation by inhibiting mTOR and caused exacerbated lung damage [15].The activation of autophagy has been considered detrimental in the airway epithelium in response to CS [16, 17]. More detailed investigations are required to explore the aberrant activation of autophagy in COPD pathogenesis.

Follistatin-like protein1(FSTL1), also known as follistatin-related protein (FRP) or transforming growth factor-1β stimulated clone 36 (TSC-36) [18], is a secreted protein involved in respiratory development and regulation of immunologic process [18–20]. FSTL1 is a novel inflammatory mediator and plays a crucial role in the regulation of inflammatory cells, which are the main characteristic of COPD. Research found that FSTL1 rose in the serum of COPD patients combined with pulmonary hypertension [21]. It was previously shown that FSTL1 played a role in autophagy, and might contribute to epithelial-mesenchymal transition (EMT) and airway remodeling in asthmatics [22, 23], but the role of FSTL1 in COPD has not been elucidated. Therefore, whether there exists an inner connection between FSTL1, autophagy and COPD needs further investigation.

## Methods

### Patients

Serum of COPD patients (n = 25) was taken from COPD patients who visited Qilu Hospital of Shandong University. Serum of control group (n = 28) was collected from subjects without COPD who visited our hospital. And lung specimens of COPD patients (n = 7) were obtained from patients who underwent pneumoresection for suspected or confirmed lung cancer. Control biopsy specimens (n = 6) with no history of COPD were obtained from Qilu Hospital Cadaver Donating Center. COPD was diagnosed according to the Global Initiative for COPD. All subjects did not have other respiratory diseases such as asthma, interstitial lung disease, silicosis pneumoconiosis, and pulmonary infection. The study has been approved by the Ethics Committee of Qilu Hospital of Shandong University, and the written informed consent has been obtained. Subject information was given in Table 1.

**Table 1:** Clinic characteristics of control subjects and COPD patients

	CON	COPD
Sex (M/F)	15/13	15/10
Age	52.214 ± 11.764	53.800 ± 9.849
FEV1/FVC	84.498 ± 8.501	53.418 ± 9.296
FEV1%, pre	92.015 ± 8.250	62.833 ± 9.690

### Mice

Age, weight, and sex-matched (8-week-old, 20 ± 3 g, female) C57BL/6 mice (6 mice each group) were obtained from the Animal Experiment Center of Shandong University (Shandong, China). Mice were retained in a pathogen-free environment at room temperature (24˚C) and controlled day/night cycles with free access to water and standard laboratory chow. C57BL/6 mice were purchased from the Experimental Animal Center of Shandong University (Jinan, China). FSTL1^flox/+^ mice were a generous gift given by Xiang Gao (Nanjing University, Nanjing, China) and Xu Zhang (Institute of Neuroscience, Shanghai Institute for Biological Sciences, Chinese Academy of Sciences, Shanghai, China). The experimental procedures were approved by the Ethics Committee of Qilu Hospital of Shandong University, China.

### Cigarette smoking and 3-methyladenine challenge

C57BL/6 mice were randomly divided into three separate groups, including air-exposed group, CS-exposed group, and CS-exposed group with 3-methyladenine intervention. Mice in the CS group were exposed to four commercially filtered cigarettes (10 mg of tar, 1.0 mg of nicotine content, 12 mg of carbon monoxide; Hademen® Filter tip cigarette) twice/day, 6 days/week for 12 weeks using a sealed chamber connected to cigarette smoke [24, 25]. The control mice were exposed to room air under the same conditions. The 3-methyladenine intervention was administered by intraperitoneal injection with 3-MA (20 mg/kg/day, Sigma, USA) for 12 weeks.

### FSTL1^±^ mice

FSTL1^±^ mice were generated as reported previously [26]. FSTL1^flox/+^ mice and FSTL1^±^ mice were randomly divided into four groups (6 each group) and exposed to cigarette smoking or room air for 12 weeks, as mentioned above.

### Mouse BALF collection

At the 24th hour after the last challenge, mice were anesthetized intraperitoneally with pentobarbital sodium (1.0%, 60 mg/kg) and euthanized by decapitation. Left lungs were lavaged three times with 1 ml of ice-cold phosphate-buffered saline (PBS) via tracheal catheter to collect BALF. After centrifugation of BALF cells, the supernatant was obtained and stored at -80˚C for subsequent experiments.

### Enzyme-linked immunosorbent assay

To detect the level of FSTL1, IL-8, TNF-α and LTB4, we tested the serum of patients and BALF of mice using enzyme-linked immunosorbent assay (ELISA) kit (CUSABIO, MD, USA) according to the manufacturer's instruction. Optical densities were measured at 450 nm and the standard curve was drawn by software to calculate the concentration.

### Western blotting

Lung tissues samples, washed with 0.01 M PBS, were lysed in ice-cold RIPA buffer containing sodium phosphate (20 mM, pH7.4), NaCl (150 mM), 1% NP-40, 0.1% SDS, and 0.5% deoxycholic acid with sodium orthovanadate (1 mM) and protease inhibitor cocktail (Roche). After centrifuging the samples at 12,000 *g* for 15 min, the supernatants were collected. The total amount of protein was quantified using the Pierce BCA Protein Assay Kit. 30 μg of total protein each were separated by 10% SDS polyacrylamide gel electrophoresis and blotted into PVDF membranes. Then the membranes were probed with primary and secondary antibodies sequentially. The primary antibodies were utilized: anti-FSTL1 (1:1000, ab71548, Abcam), anti-collagen I (1:1000, BA2023, BOSTER), anti-SMA (1:1000, BA0002, BOSTER), anti-P62 (1:1000, ab56416, Abcam), anti-LC3B (1:1000, ab48394, Abcam), and anti-GAPDH (1:1000, BA2913, BOSTER). The densitometry of bands was performed by ImageJ software (version 1.46).

### Lung morphometry

Lung tissues samples were fixed in a 10% solution of formaldehyde for 24 h. Then, samples were embedded in paraffin and cut into 4 μm slices, which were dewaxed and rehydrated before HE staining. After staining, slides were dehydrated, mounted with neutral gum and observed under a microscope. Mean linear intercept (MIL) was measured from the stained mice lung sections. 10 random 40 × fields from each animal were calculated by direct estimation of MIL based on intercept distribution [27].

### Immunohistochemistry

Immunohistochemistry was performed after dewaxing and rehydration. Lung slices were pretreated with an EDTA-antigen retrieval buffer in a microwave oven, and endogenous peroxidase in tissue was quenched with 3% H_2_O_2_ for 10 min. Lung slices were blocked with 20% normal fetal bovine serum for 30 min at 37 °C, then incubated with primary and secondary antibodies. The primary antibodies were utilized: anti-FSTL1 (1:200, ab71548, Abcam), anti-collagen I (1:200, BA2023, BOSTER), anti-SMA (1:400, BA0002, BOSTER), anti-P62 (1:200, ab56416, Abcam), anti-LC3B (1:200, ab48394, Abcam), and anti-GAPDH (1:400, BA2913, BOSTER). The staining was performed using the PV-9000 kit (Zhongshan Golden Bridge Biotechnology Co, China). After dehydrating and mounting, slices were observed under a microscope. For the expression of each protein, three slices of IHC were used to calculate the average optical density (AOD) per sample. Quantification of IHC was also performed using ImageJ.

### Transmission EM

Lung tissues samples were fixed with 2.5% glutaraldehyde and postfixed with 1% osmium tetroxide in sodium cacodylate buffer for 2 h at 4 °C. Then the samples were stained with 1% Millipore-filtered uranyl acetate, dehydrated in graded ethanol and propylene oxide. After being embedded in LX-112 medium, samples were thin sectioned (70 nm) using an ultra-cut microtome (Leica). Ultrathin sections were stained with 4% uranyl acetate and lead citrate, and sections were examined using an electron microscope (JEM).

### Assessment of lung function

Lung function was assessed with FlexiVent ventilator (SCIREQ, Canada) for small animals when mice were stable. At the 24th hour after the final exposure, mice were anesthetized intraperitoneally and tracheostomized. After intubation, mice were mechanically ventilated to assess lung function. Using pressure–volume (PV) loops, lung function was measured including total lung capacity (TLC) and lung compliance (chord compliance, Cchord).

### Statistics analysis

Data were presented as mean ± SD. Student’s t-test and ANOVA were used to evaluate differences in measured variables for group comparisons (at least 3 independent experiments). Statistical analysis was conducted with SPSS 20.0 and GraphPad Prism 5.0, and significance was defined at P < 0.05.

## Results

### FSTL1 expression and autophagy activation were elevated in COPD patients

**Fig. 1:**
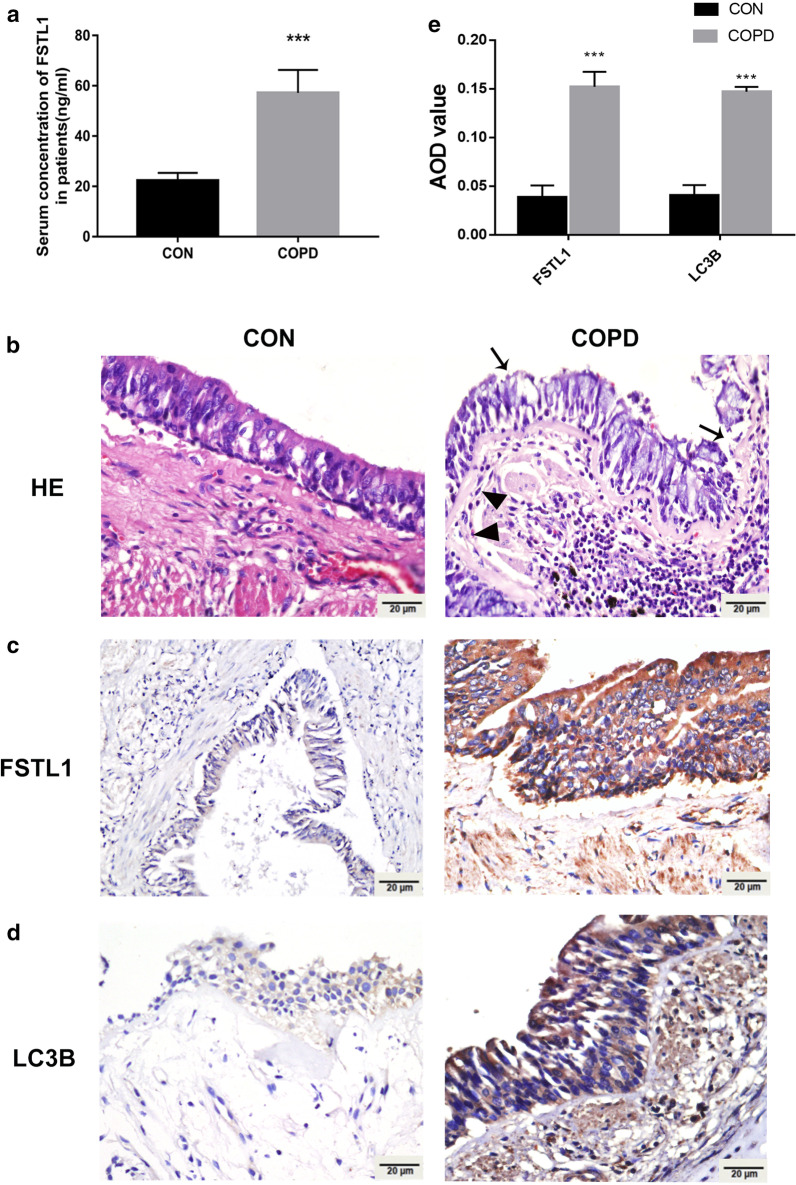
FSTL1 was highly expressed in COPD patients and correlated with autophagy. **a** FSTL1 level was significantly higher in the serum of COPD patients (n = 28) compared with control (n = 25) (ELISA). **b** Airway specimens of COPD showed inflammation infiltration, RBM fragmentation (black triangle), epithelial integrity loss, and cilia lodging (black arrow) (HE staining, ×400). **c** Positive areas of FSTL1 in airway specimens of COPD patients were significantly larger than those of control subjects (immunohistochemistry staining, ×400). **d** Positive areas of LC3B in airway specimens were significantly larger than control subjects (immunohistochemistry staining, ×400). **e** Quantification of IHC. Median of each group is presented. * difference between control and COPD group, p < 0.05; ***p < 0.001, t-test. Scale bar, 20 μm. Abbreviations: CON, control

Our previous study has shown that FSTL1 may promote EMT and airway remodeling in asthma by activating autophagy. However, it remains unclear whether FSTL1 plays a role in other chronic respiratory diseases, like COPD. In this study, we found increased circulating FSTL1 in the serum of COPD patients compared with control subjects (Fig. 1a). Airway specimens of COPD patients (Fig. 1b) showed inflammation infiltration, RBM fragmentation, epithelial integrity loss, and cilia lodging by HE staining. Meanwhile, positive areas of FSTL1 and LC3B staining in epithelial cells and mesenchyme were significantly larger than those in control subjects by immunohistochemistry staining (Fig. 1c–e). These results indicated that FSTL1 was highly expressed in COPD patients with autophagy activation.

### FSTL1 expression in mice was increased in response to CS exposure

**Fig. 2:**
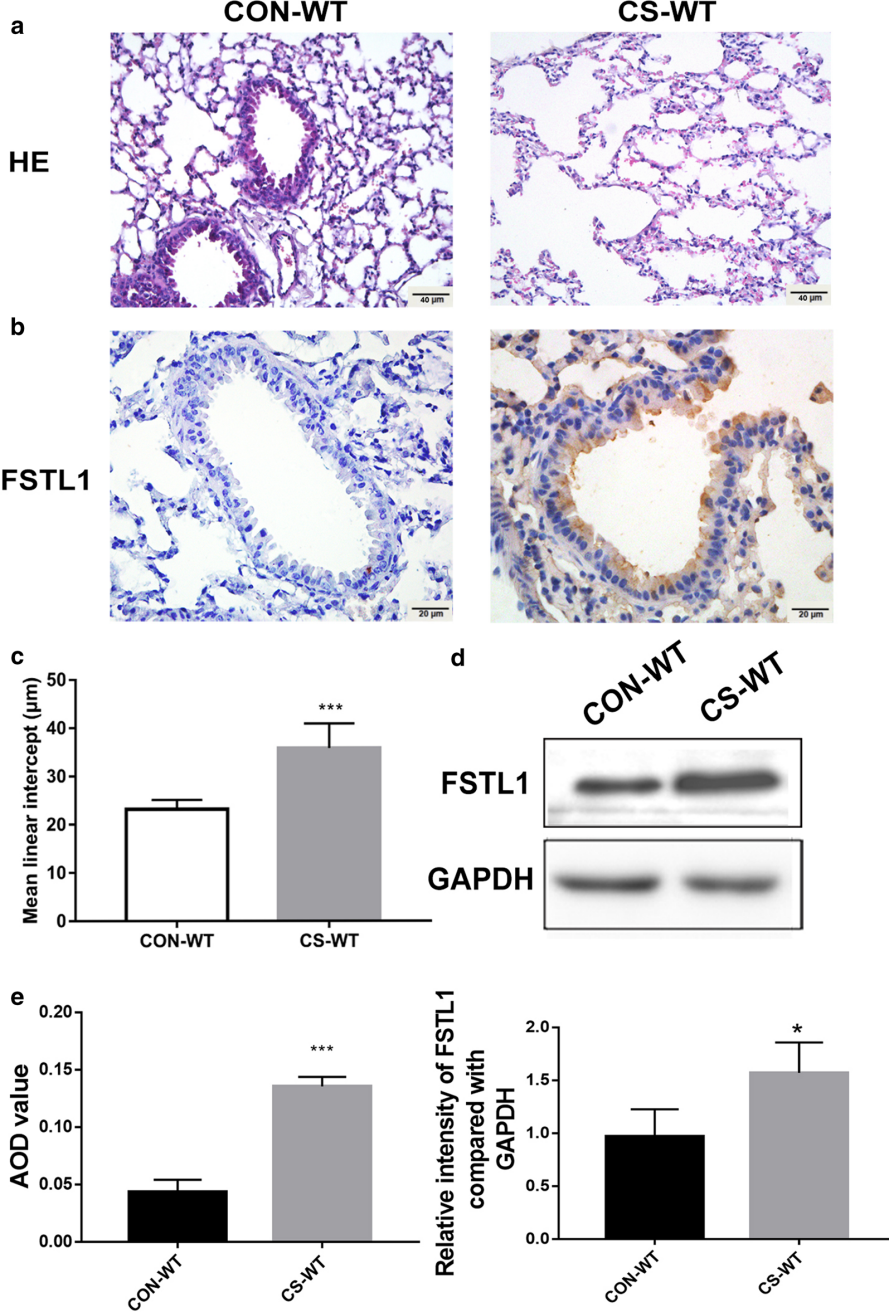
CS-exposed mice exhibit higher expression of FSTL1. **a**, **c** The lung tissues of CS-WT mice showed an active inflammatory process, small airway obstruction and augmented airspace (HE staining, ×200. Scale bar, 40 μm). Mean linear intercept was measured (10 random fields each mouse). **b** Positive areas of FSTL1 in lung tissues of CS-exposed mice were significantly larger than those in control group (immunohistochemistry, ×400. Scale bar, 20 μm). **d** The protein level of FSTL1 in the lung of CS-WT mice is higher than control group (western blotting). **e** Quantification of IHC and WB. Each group consisted of 6 mice, and the median of each group is presented. All the experiments were repeated independently at least 3 times. *difference between CON-WT and CS-WT, p < 0.05; ***p < 0.001; t-test. Abbreviations: CS, cigarette smoke. WT, wild type. ANOVA, analysis of variance

To investigate the correlation between FSTL1 and the pathogenesis of COPD, we generated a CS-induced mouse model. By performing HE staining and measurement of MLI, the lung specimen of CS-exposed mice showed an active inflammatory response and emphysema process, including destruction of lung parenchyma and alveolar walls (Fig. 2a, c). Western blotting and immunohistochemistry staining were carried out to confirm FSTL1 expression after CS exposure. Results showed that epithelial cells and mesenchyme demonstrated positive staining of FSTL1 (Fig. 2b, e) in CS-exposed mice. Consistently, western blotting using lung homogenates showed a higher level of FSTL1 with CS exposure (Fig. 2d, e). These results suggest that cigarette smoking may upregulate the expression of FSTL1 in lung tissues, especially in epithelial cells.

### CS exposure activates autophagy, promoting airway remodeling and airway inflammation

**Fig. 3:**
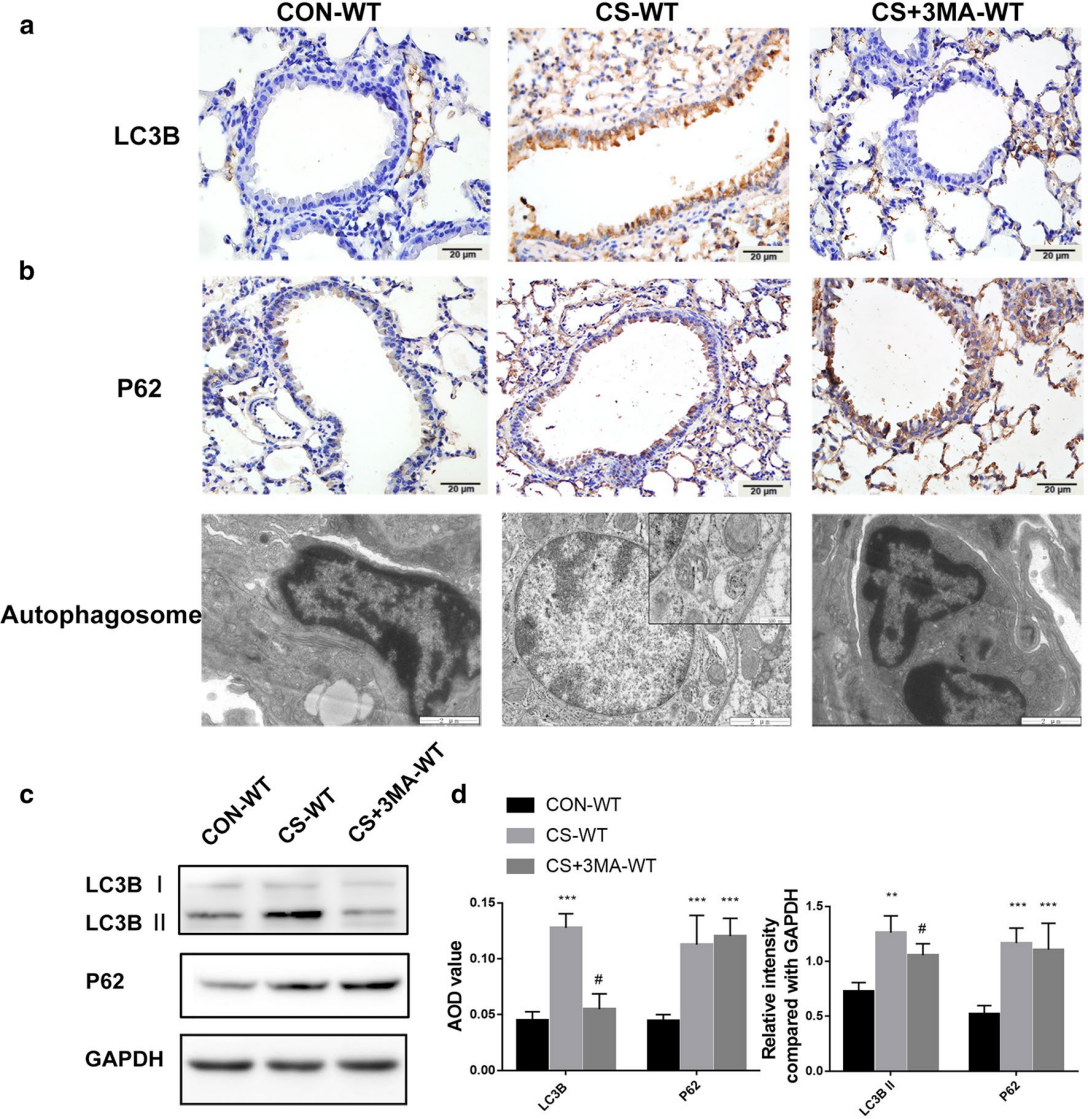
Dysregulation of autophagy in CS-exposed mice. **a** Positive areas of LC3B and P62 in lung tissues of CS-WT mice were significantly larger than those in control group, and injection with 3-MA lightened the differences. (immunohistochemistry, ×400. Scale bar, 20 μm). **b** Autophagosomes formation was enhanced in CS-WT mice and alleviated by 3-MA pretreatment (transmission electron microscopy. Scale bar, 2 μm,500 nm). **c** The protein level of LC3B and P62 in each group (Western blotting). **d** Quantification of IHC and WB. Each group consisted of 6 mice, and the median of each group is presented. All the experiments were repeated independently at least 3 times. *difference compared with CON-WT, p < 0.05; **p < 0.01; ***p < 0.001; ^#^difference between CS-WT and CS + 3MA-WT, p < 0.05; one-way ANOVA, t-test. Abbreviations: CS, cigarette smoke. WT, wild type. 3-MA, 3-methyladenine. ANOVA, analysis of variance

**Fig. 4:**
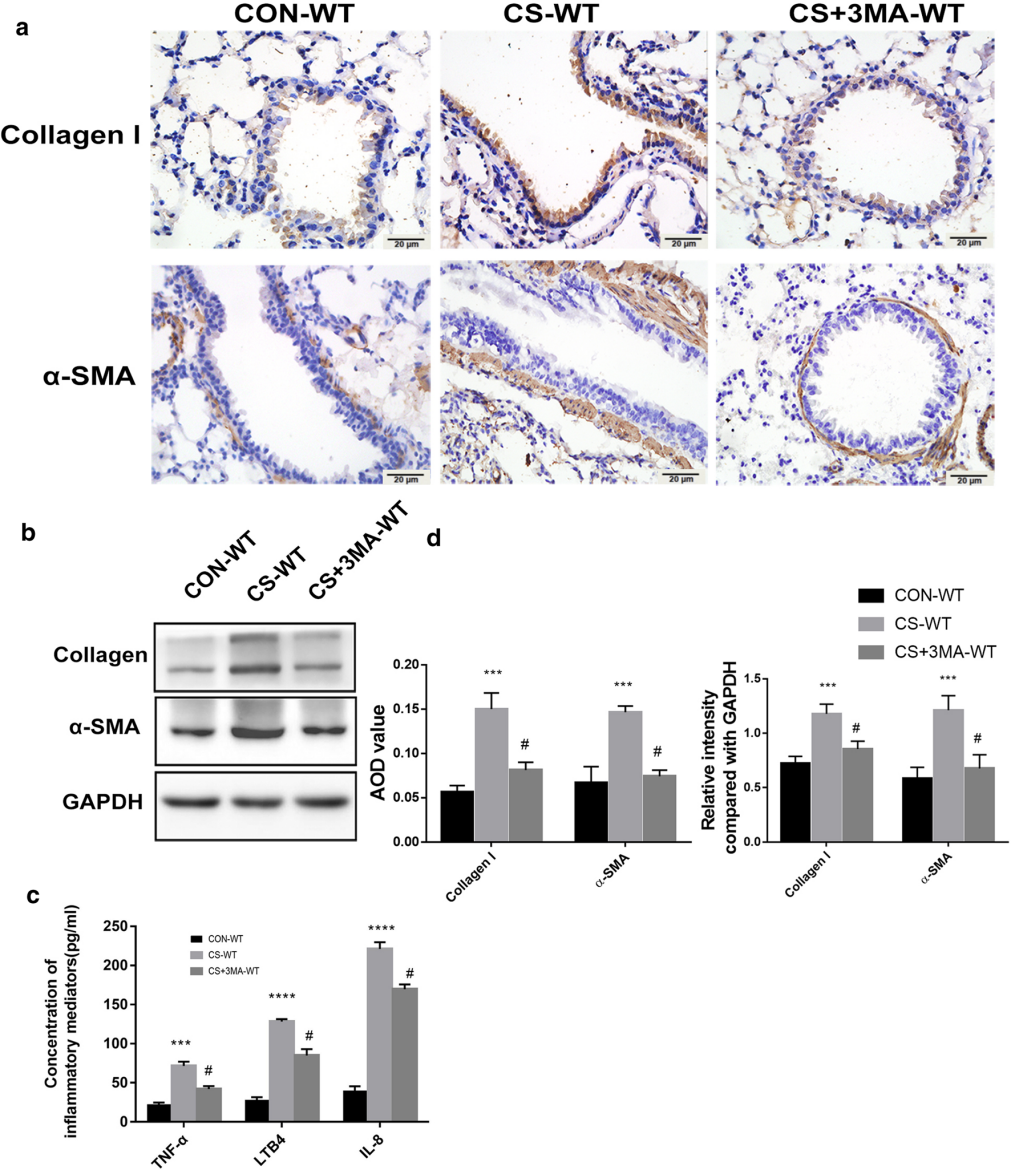
CS-induced autophagy leads to airway remodeling and airway inflammation. **a** Positive areas of collagen I and α-SMA in lung tissues of CS-WT mice were significantly larger than control group, and 3-MA injection lightened airway remodeling (Immunohistochemistry, ×400x). **b** The protein level of collagen I and α-SMA in each group (Western blotting). **c** Levels of TNF-α, IL-8 and LTB4 increased significantly in BALF of CS-WT mice and 3-MA injection alleviated the change (ELISA). **d** Quantification of IHC and WB. Each group consisted of 6 mice, and the median of each group is presented. All the experiments were repeated independently at least 3 times. *difference compared with CON-WT, p < 0.05; **p < 0.01; ***p < 0.001; ^#^difference between CS-WT and CS + 3MA-WT, p < 0.05; one-way ANOVA, t-test. Scale bar, 20 μm. Abbreviations: CS, cigarette smoke. WT, wild type. 3-MA, 3-methyladenine. BALF, bronchoalveolar lavage fluid. ANOVA, analysis of variance

To elucidate the regulatory role of FSTL1 in CS-exposed mice, we detected the biomarkers of airway remodeling, airway inflammation and autophagy in mice models and found upregulated LC3B in lung parenchyma and small airway (Fig. 3a), as well as increased LC3B II/I ratio and autophagosome formation in airway epithelial cells by transmission electron microscopy (Fig. 3b, c), which proved activated autophagy. P62 also increased in CS-exposed mice, indicating insufficient autophagic clearance (Fig. 3a). As expected, collagen I and α-SMA were highly expressed in airway specimens of CS-exposed mice (Fig. 4a). Western blotting analysis of lung total protein was performed, and a consistent conclusion was attained (Figs. 3c, d, 4b, d). Certain inflammatory cytokines were measured using ELISA to study airway inflammation. As shown in Fig. 4c, levels of TNF-α, IL-8 and LTB4 increased significantly in BALF of CS-exposed mice. These results indicate that FSTL1 may promote CS-induced airway remodeling and airway inflammation by regulating autophagy.

### Inhibit autophagy with 3-MA can attenuate airway remodeling and inflammation in CS-exposed WT mice

To further explore the effect of autophagy on COPD, CS-exposed mice were treated with 3-methyladenine (3-MA), a chemical inhibitor of PI3K. Under transmission electron microscopy, the formation of autophagosome in epithelial cells was markedly inhibited using 3-MA pretreatment (Fig. 3b). Furthermore, immunohistochemistry staining and western blotting analysis showed a decreased level of LC3B and an increasing level of P62 after 3-MA pretreatment (Fig. 3a, c, d). These results suggest that autophagy in CS-exposed mice was suppressed by 3-MA. Meanwhile, compared with CS-exposed mice, the 3-MA pretreated mice exhibited lower expression of collagen I, α-SMA (Fig. 4a, b, d) and inflammatory mediators mentioned before (Fig. 4c). Our investigation proved that inhibiting autophagy with 3-MA can attenuate CS-induced airway remodeling and inflammation in WT mice.

### FSTL1 deficiency attenuates autophagy activation in response to CS exposure

**Fig. 5:**
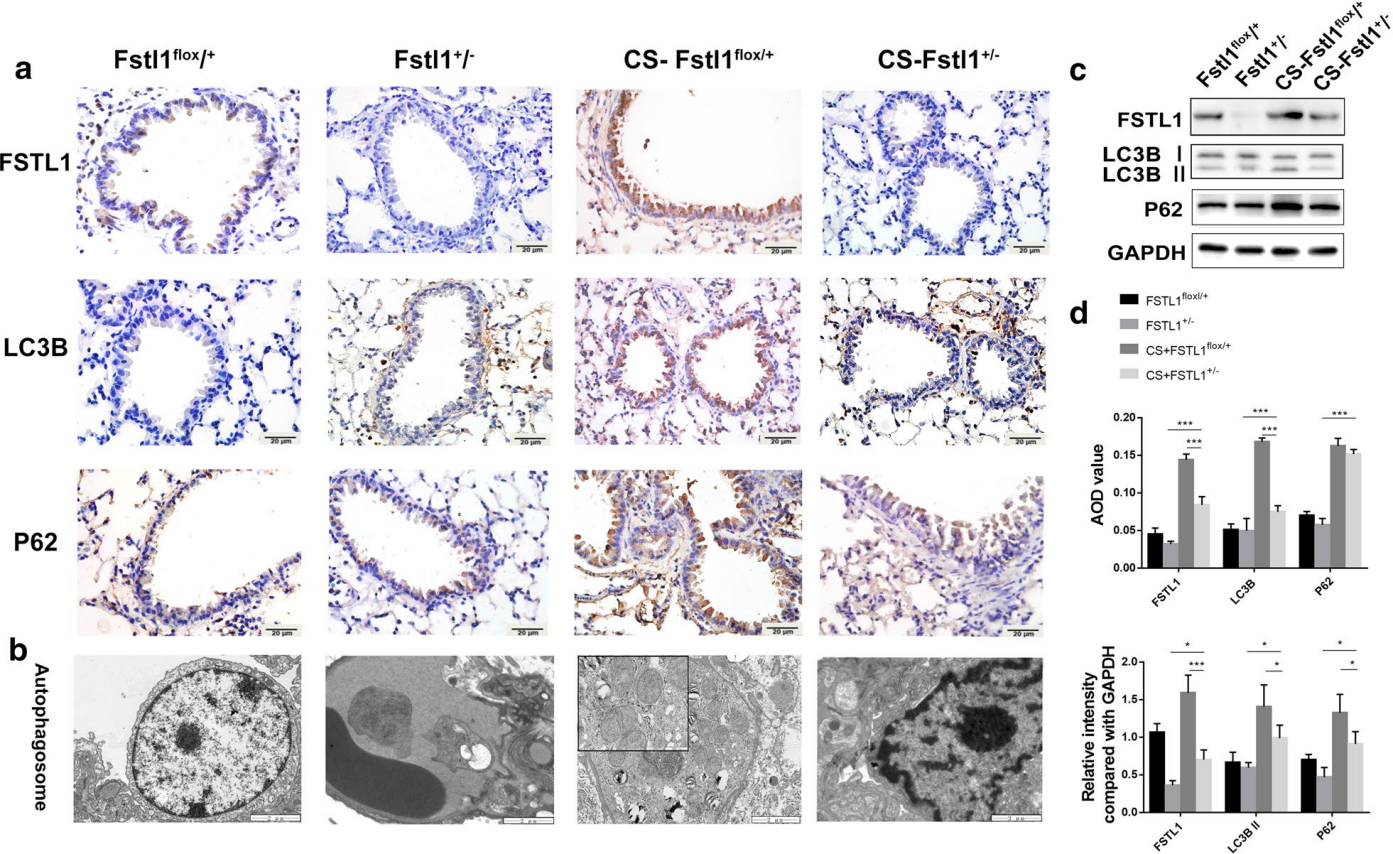
The haplodeletion of FSTL1 attenuates autophagy in CS-exposed model. **a** The staining of FSTL1 and autophagic proteins LC3B, P62 in lung tissues of FSTL1^±^ and FSTL1^flox/+^ mice with or without CS exposure (Immunohistochemistry, ×400. Scale bar, 20 μm.). **b** Autophagosome formation was inhibited in CS-FSTL1^±^ mice. (Transmission electron microscopy. Scale bar,2 μm,500 nm). **c** The protein level of FSTL1, LC3B and P62 in each group (Western blotting). **d** Quantification of IHC and WB. Each group consisted of 6 mice, and the median of each group is presented. All the experiments were repeated independently at least 3 times. *p < 0.05; **p < 0.01; ***p < 0.001; one-way ANOVA, t-test. Abbreviations: CS, cigarette smoke

In WT mice model, we found that FSTL1 and autophagy may play a role in the development of COPD. To further examine the effect of FSTL1 on autophagy regulation and COPD pathogenesis, an animal model was prepared with FSTL1^±^ mice and FSTL1^flox/+^ mice (Fig. 5a). After 12 weeks of exposure to cigarette smoke or room air, we detected the level of FSTL1, LC3B, P62 and autophagosome in an animal model using immunohistochemistry staining and western blotting analysis. As predicted, LC3B II conversion (Fig. 5c, d) and autophagosome formation (Fig. 5b) were increased after CS exposure. However, these changes were reduced in CS-FSTL1^±^ mice than CS-FSTL1^flox/+^ mice, illustrating that haplodeletion of FSTL1 attenuated CS-induced autophagy activation. The most significant elevation of P62 was observed in CS-FSTL1^flox/+^ mice, and haplodeletion of FSTL1 also mitigated the effect of CS exposure (Fig. 5a, c, d). According to these, FSTL1 deficiency can not only protest against CS-induced autophagy activation but also improve autophagic clearance.

### FSTL1 deficiency relieves airway remodeling and inflammation in response to CS exposure

**Fig. 6:**
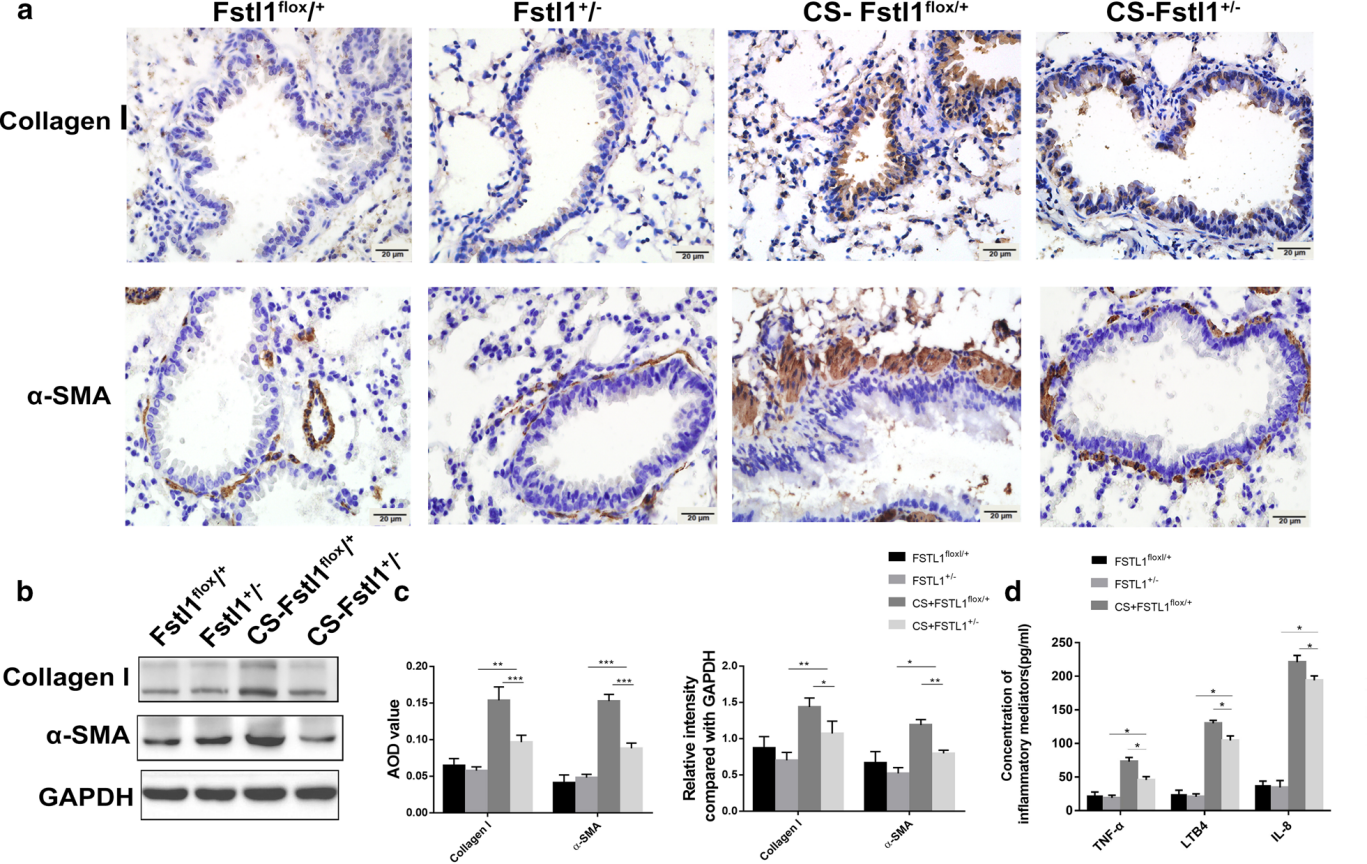
FSTL1 deficiency abates airway remodeling and airway inflammation. **a** Positive areas of collagen I and α-SMA in lung tissues of FSTL1^±^ and FSTL1^flox/+^ mice with or without CS exposure (immunohistochemistry, ×400). **b** The protein level of collagen I and α-SMA in each group (western blotting). **c**: Quantification of IHC and WB. **d** Levels of TNF-α, IL-8 and LTB4 increased significantly in BALF of CS-FSTL1^flox/+^ mice than CS-FSTL1^±^ mice (ELISA). Each group consisted of 6 mice, and the median of each group is presented. All the experiments were repeated independently at least 3 times. *p < 0.05; **p < 0.01; one-way ANOVA. Scale bar, 20 μm. Abbreviations: CS, cigarette smoke. BALF, bronchoalveolar lavage fluid

Compared with FSTL1^flox/+^ mice, there was a more subdued increase of collagen I and α-SMA in FSTL1^±^ mice after CS exposure by immunohistochemistry staining (Fig. 6a, c) and western blotting analysis (Fig. 6b, c). Meanwhile, after CS exposure, the increase of IL-8, LTB4 and TNF- α in BALF of FSTL1^±^ mice was lower than that in FSTL1^flox/+^ mice (Fig. 6d), indicating that airway inflammation was alleviated. These results demonstrate that knockdown of FSTL1 alleviates CS-induced airway remodeling and inflammation in mice.

### Both FSTL1 deficiency and autophagy inhibitor rescue lung function decline in CS-exposed mice

**Fig. 7:**
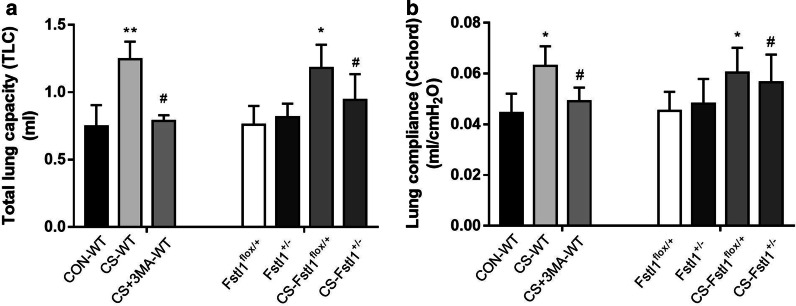
FSTL1 deficiency and autophagy inhibitor both alleviate lung function decline in CS-exposed mice. **a** After CS exposure, total lung capacity did not increase in the 3-MA pretreatment mice, and FSTL1^±^ mice showed a similar protective effect as 3-MA versus FSTL1^flox/+^ mice. **b** After CS exposure, lung compliance did not increase sharply in the 3-MA pretreatment mice, and FSTL1^±^ mice showed a similar protective effect as 3-MA versus FSTL1^flox/+^ mice. Each group consisted of 6 mice, and the median of each group is presented. All the experiments were repeated independently at least 3 times. *difference between CON-WT and CS-WT, FSTL1^flox/+^ and CS-FSTL1^flox/+^, p < 0.05; **p < 0.01; ^#^difference between CS-WT and CS + 3MA-WT, CS-FSTL1^±^ and CS-FSTL1^flox/+^, p < 0.05; one-way ANOVA. Abbreviations: CS, cigarette smoke. 3-MA, 3-methyladenine. CON, control. WT, wild type

To verify the effect of FSTL1 and autophagy on the lung function of CS-exposed mice, total lung capacity and lung compliance were measured using a ventilator for small animals after the final exposure. Both 3-MA pretreated WT mice and FSTL1^±^ mice showed attenuated lung function decline in response to CS exposure. Hyperinflation occurred in CS-WT mice and CS-FSTL1^flox/+^ mice as the total lung capacity (TLC) and lung compliance increased after chronic CS exposure. However, TLC (Fig. 7a) and Cchord (Fig. 7b) did not rise significantly in the 3-MA pretreated WT mice and FSTL1^±^ mice after CS exposure. The data suggest that FSTL1 deficiency can protest against the decline of lung function after CS exposure as autophagy inhibitor did.

## Discussion

The development of COPD involves inducible responses to inhaled particles, including cigarette smoke, and stress triggered by cigarette smoke exposure can induce autophagy in lung tissues. In this study, we report that FSTL1 is highly expressed in COPD patients together with autophagy activation, airway inflammation and airway remodeling. In both COPD patients and CS-exposed animal model, we found the level of FSTL1 was positively related to overactive autophagy. Moreover, autophagy inhibition with 3-MA can attenuate airway remodeling and inflammation to a certain extent in CS-exposed mice. By establishing FSTL1^±^ mice model, it was proved that the haplodeletion of FSTL1 had a repressive effect on autophagy, indicating that FSTL1 might play a vital role in the activation of autophagy. In conclusion, our study has found a link between FSTL1, autophagy and the pathogenesis of COPD, supporting our hypothesis that FSTL1 modulates CS-induced autophagy and plays a role in the COPD process.

FSTL1 is implicated in various biological and pathological processes, including apoptosis, autophagy, proliferation, differentiation and migration [19]. Generated mainly by mesenchymal cells (fibroblasts, chondrocytes, osteocytes, etc.), FSTL1 affects immunomodulation, tumorigenesis, fibrogenesis and organ development via multiple signal pathways [28]. As an antagonist of the BMP4 signaling pathway, FSTL1 plays a crucial part in the embryogenesis of lung and alveolar, thus FSTL1^−/−^ mice showed postnatal lethality due to respiratory failure [29, 30]. There is growing evidence that FSTL1 plays an important role in respiratory diseases. Recent studies prove that FSTL1 participates in lung injury as it is secreted in response to bleomycin, silica, radiation and other lung injury inducers [30–32]. Besides, FSTL1 has an association with airway remodeling in chronic airway diseases [33]. Our previous study suggested that FSTL1 was positively linked to thickened RBM and smooth muscle mass in asthmatics. Moreover, FSTL1 can induce EMT and airway remodeling in asthma [34]. Several FSTL1 SNPs were found corresponding to COPD and lung function, and FSTL1 deficiency might protect mice from cigarette smoke-induced emphysema [35]. In today’s study, we further unravel the role of FSTL1 in the pathogenesis of COPD. The increased level of FSTL1 in COPD is tied to overactive autophagy, aggravating airway remodeling and airway inflammation. Besides, haplodeletion of FSTL1 in CS-exposed mice abate airway remodeling and airway inflammation through inhibiting CS-induced autophagy.

Autophagy is a lysosome-dependent degradation of microorganisms, cellular proteins and organelles in order to maintain the homeostatic balance, consisting of macroautophagy, microautophagy and selected autophagy [36, 37]. LC3B (microtubule-associated protein 1 light chain 3 beta), as one of autophagy-related (ATG) proteins, represents autophagosome formation by the conversion from LC3B-I to LC3B-II [38, 39]. Z.H. Chen et al.reported that LC3B exerted a critical pro-pathogenic role in the development of CS-induced emphysema [40]. P62, also known as sequestosome 1 (SQSTM1), which is directly conjugated to LC3B, is self-degraded by autophagy and indicates insufficient autophagic clearance [41, 42]. Our data suggest a higher LC3B II/I ratio and P62 expression in CS-exposed animal model. Also, the autophagosome formation was more obvious after CS exposure. These results revealed that CS triggered overactive autophagy and insufficient autophagic clearance in the lung. Meanwhile, pretreatment with 3-MA dramatically reduced CS-induced autophagy and disease progression. It is well known that autophagy plays a critical role in pulmonary inflammation and pathogenesis of numerous chronic lung diseases [13]. However, in COPD which undergoes prolonged inflammation and stress, the function of autophagy is more complicated.

There is increasingly more evidence that dysregulated autophagy is related to the pathogenesis of COPD. Some studies suggest that autophagy contributes to COPD progress. Increased autophagic proteins were found in lung tissues from COPD patients [43]. In vitro, autophagy was considered as an early event in COPD progression [43]. CS-induced autophagy in epithelial cells promotes the production of TNF-α, IL-6 and IL-8 and lymphocyte recruitment into the lung [44]. It appears that CS initiates and prompts airway inflammation by increasing autophagosomal turnover (flux) and further results in epithelial cell apoptosis and death [16, 17, 40, 43, 45].Moreover, recent advances deem autophagy has a potential impact on airway fibrosis [46]. Deficiency of autophagy alleviates CS-induced cilia shortening in vitro and reduces profibrotic signaling pathway and ECM release *in vitro* [45]. In vivo experiment, we validated that inhibition of autophagy activation with 3-MA mitigated CS-induced airway remodeling and airway inflammation, further enriching evidence that overactive autophagy might exacerbate COPD progression. On the other hand, there are other mechanisms of dysregulated autophagy in COPD. Functional autophagy is critical to degrade damaged organelles and proteins and maintain homeostasis. Some research indicate that autophagy impairment induced by ROS from CS and mitochondria can accelerate lung aging and emphysema exacerbations [47]. Inadequate autophagy can induce senescence in COPD and also contribute to the development of idiopathic pulmonary fibrosis [48, 49]. Autophagy augmentation appears to be a therapeutic target for alleviating aging and COPD progress [50]. In our study, P62 increased after CS exposure, suggesting impaired autophagy while overactive autophagy was found. Dysregulated autophagy in COPD pathogenesis may operate in two extremes. Since autophagy is a well-known dynamic and complicated process, innovative methods and specific regulators should be developed to investigate autophagy in “real-time”.

In our research, FSTL1 was elevated together with autophagosome formation and autophagic proteins in both COPD patients and CS-exposed animal model. Pretreatment with autophagic inhibitor in WT mice could alleviate CS-induced airway inflammation, airway remodeling and impaired lung function. In FSTL1^±^ mice, the deficiency of FSTL1 also bated CS-induced autophagy activation and the adverse response to CS in lung tissues. These results indicate that FSTL1 may modulate autophagy by certain signaling pathways in COPD. From the existing study, we can learn that FSTL1 have crosstalk with several autophagic signaling pathways. Via DIP2A receptor, FSTL1 can activate the Akt pathway and finally attenuate apoptosis after MCAO in Rats [51]. In cardiovascular diseases, FSTL1 has been implicated in the activation of the PI3K/Akt signaling, exhibiting protective effects [52–54]. Furthermore, FSTL1 is involved in the activation of the AMPK signaling pathway in cardiac and renal diseases [55, 56]. These studies shed light on the correlation between FSTL1 and the regulation of autophagy. However, there are some limitations. The present study cannot demonstrate a complete mechanism of FSTL1, dysregulated autophagy and COPD. Additional work is needed, including in vitro experiments with more accurate autophagic regulators, to study autophagy and investigate the mechanism of how FSTL1 exerts the effect on autophagy and leads to COPD.

## Conclusions

In this study, it was proved that FSTL1 could promote the CS-induced COPD process by regulating autophagy. In conclusion, our study demonstrates the important role of FSTL1 and autophagy in COPD occurrence and progression, providing potential avenues for therapeutic targeting of COPD.

## Data Availability

The datasets used and/or analysed during the current study are available from the corresponding author on reasonable request.

## Abbreviations

CS: Cigarette smoke
COPD: Chronic obstructive pulmonary disease
FSTL1: Follistatin-like protein 1
WT: Wild-type
3-MA: 3-Methyladenine
EMT: Electron microscope technology
PBS: Phosphate buffered saline
ELISA: Enzyme-linked immunosorbent assay
MIL: Mean linear intercept
TLC: Total lung capacity
Cchord: Chord compliance
ATG: Autophagy-related proteins
LC3B: Microtubule associated protein 1 light chain 3 beta
BALF: Bronchoalveolar lavage fluid
